# Intratumoral levels and prognostic significance of *Fusobacterium nucleatum* in cervical carcinoma

**DOI:** 10.18632/aging.104188

**Published:** 2020-11-14

**Authors:** Shu-Ting Huang, Jing Chen, Li-Yin Lian, Hui-Hua Cai, Hai-Shan Zeng, Min Zheng, Mu-Biao Liu

**Affiliations:** 1 Department of Gynecology, Guangdong Provincial People’s Hospital, Guangzhou, P. R. China; 2 Guangdong Academy of Medical Sciences, Guangzhou, P. R. China; 3 Department of Gynecology, Sun Yat-Sen University Cancer Center, Guangzhou, P. R. China

**Keywords:** *F. nucleatum*, cervical cancer, prognosis, CSCs, recurrence and metastasis

## Abstract

Growing evidence suggests that microbes can influence the onset of cancer and its consequent development. By researching samples from patients afflicted by cervical cancer, we aimed to explore the associated dynamics and prognostic value of intratumoral levels of *F. nucleatum*. We used qPCR to analyze tumor tissues obtained from 112 cervical cancer patients in order to characterize the levels and influences of intratumoral levels of the *F. nucleatum*. Especially for recurrent tissues, there was a distinct observation of higher levels of *F. nucleatum* in cervical cancer. Patients with high burdens of *F. nucleatum* intratumoral infiltration exhibited correspondingly poor rates of both overall survival and progression-free survival. Measures of the levels of *F. nucleatum* were found to have been reliable independent prognostic factors that could predict rates of PFS for afflicted patients (HR = 4.8, 95%CI = 1.2-18.6, *P* = 0.024). Notably, the levels *of* *F. nucleatum* were positively correlated with tumor differentiation. Cancer cells from patients with relatively high levels of *F. nucleatum* were observed to possess the characteristics of cancer stem cells (CSCs). We propose that *F. nucleatum* might be one potential cervical cancer diagnostic and prognostic biomarker, and these findings will help to provide a sound rationale and merit for further study of this bacterium.

## INTRODUCTION

Despite advancements in screening techniques as well as a wider availability of vaccines, cervical carcinoma continues to be the most common cancer afflicting women globally [1]. Accordingly, there is a high rate of incidence and in the year 2019 in the United States there were greater than 13,100 new diagnoses, which led to greater than 4,250 deaths [2]. Radical forms of surgery and radiotherapy are potential curative treatment options for patients diagnosed with early-stage cervical cancer [3]. However, approximately 10% of early-diagnosed patients will experience recurrence or metastasis within five years of initial diagnosis and as a result, the overall prognoses for such types of afflicted patients remains poor [4]. Overall, metastasis and recurrence are the major causes for treatment failure for these patients. Thus, there is a need for deeper insights into the mechanistics and dynamics underlying cervical cancer afflictions. Such insights should be expected to help better guide as well as improve upon existing therapeutic regimes, while also potentially informing and leading to the development of novel treatments.

Several laboratory based research endeavors have reported that microbiota in tumor microenvironments contribute to the onset of and progression of cancer [5, 6]. For example, bacterial-induced inflammation has been linked with the promotion and corresponding progression of cancers via indirect distal effects from the gastro intestinal tract microbiome and likewise in a more direct manner such as in the case of influences of *Helicobacter pylori* [7–9]. Research has revealed that relatively harmful gut bacteria may indirectly impact prognoses for patients with colon cancer via influencing outcomes of chemotherapy treatments by promoting extinction of anticancer Th17 immune cells [6, 10]. Indeed, via releases of endogenous enzymes, bacteria have the capacity to transform organic chemicals, such as nutrients, pollutants, drugs, and other organic molecules. Such transformations are exemplified by the uses of bacteria in the field of industrial biotransformation in which various taxa are used to chemically modify non-biological organic molecules and thereby modulate their degradation [11, 12]. Conceivably, there is a potential for direct interactions between the outcomes of treating tumors and varied types of microbiota associated with various types of tissues, organs, and sites in the human body.

*Fusobacterium nucleatum* (*F.nucleatum*; a non-spore-forming, anaerobic gram-negative bacterium) has been reported to be frequently present in the human oral cavity and oral microbiome, as well as in the gastrointestinal and genital tracts [13], and it has already been suggested that *F. nucelatum* acts as a pathogen in the dynamics underlying gastrointestinal cancer disease [14–16]. *F. nucleatum* has revealed an overabundance in esophageal squamous carcinoma [17] and colorectal cancers [15, 16, 18] and high levels of this bacterium were positively correlated with poor prognoses for patients afflicted with these cancers. Evidence provided from an assessment of colorectal cancer by Rubinstein et al indicated that *F. nucleatum* leads to the onset and progression of colorectal cancer and associated cell proliferation [19]. In a study by Yamamura et al, patients with relatively higher levels *of* *F. nucleatum* had worse measures of RFS in esophageal squamous carcinoma [20]. However, the potential role of *F. nucleatum* in genital tract malignant tumors also remains to be fully elucidated.

Therefore, we sought to investigate intratumoral levels and burdens of *F. nucleatum* present in the vaginal microenvironment as this is a bacteria species with the potential to influence prognoses for patients afflicted by cervical cancer. In addition, we hoped to clarify differences in levels and burdens of *F. nucleatum* between cervical cancer and its recurrent lesions. Ultimately, we hoped to provide important and needed novel groundwork meriting further research aimed at elucidating the dynamics underlying *F. nucleatum* and its associated potential to indicate risk of the onset and progression of cervical cancer and sought to elucidate what new and or improved treatment options might be possible.

## RESULTS

### *F. nucleatum* abundances at higher levels especially in advanced stage and relapsed disease

**Figure 1:**
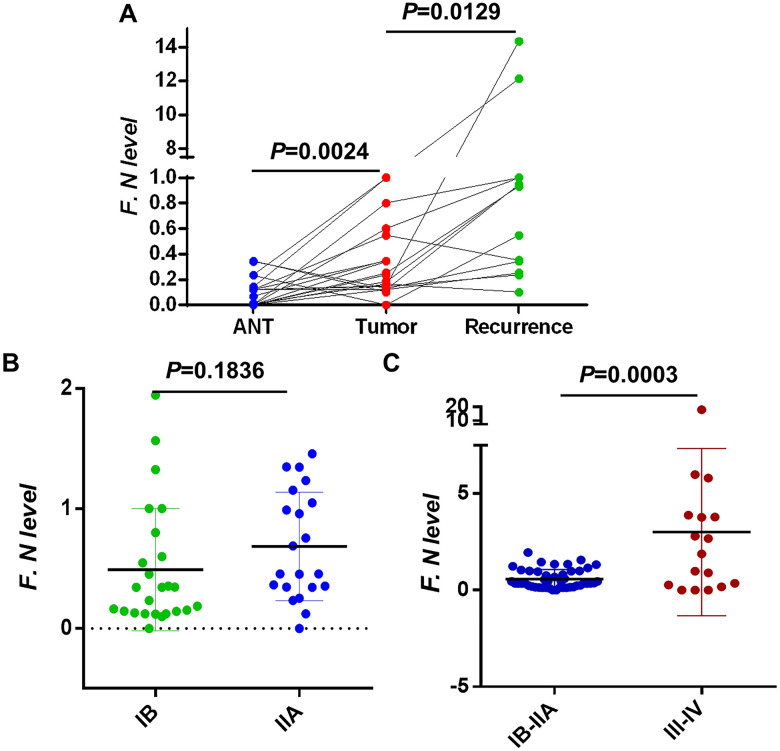
**Intratumoral *F. nucleatum* levels in cervical cancer tissues.** (**A**) F. nucleatum levels in 23 pairs of (Left) adjacent non-tumor tissues (ANT) vs. cervical cancer and (Middle) cervical cancer vs. (Right) recurrent cervical cancer tissues. (**B**) The relative amount of *F. nucleatum* in 23 cervical cancer tissues within I/II stage and (**C**) 20 advanced cancer tissues III/IV. Differences were assessed with a paired two-tailed t-test. F.N, F. nucleatum

We collected primary cancer tissues and matched non-tumorous tissues and recurrent tumor specimen from these in individual 23 relapsed patients. Firstly, we evaluated the levels of *F. nucleatum* in cervical cancer tissues by using quantitative polymerase chain reaction (qPCR) assays, and concurrently analyzed these in individual patient matched non-tumorous tissues. Results from the assessment of all 23 patients indicated that burdens of *F. nucleatum* quantified by the orthonormal -^ΔΔ^Ct were significantly higher (*P* = 0.0024, Figure 1A) in tumor tissues compared with the adjacent non-tumor tissues. The results from paired tissue assessments for individual patients likewise revealed higher *F. nucleatum* levels in 17/23 pairs. We analyzed the abundance of *F. nucleatum* in relapsed patients above 23 all patients, and observed a marked enrichment in recurrent lesion than primary cervical cancer tissues (*P* = 0.0129, Figure 1B). The abundance of *F. nucleatum* based upon all tumor stages was analyzed. We found no difference in *F. nucleatum* levels in locally advanced cervical cancer tissues (IB, n = 25/IIA, n = 21; *P* = 0.1836, Figure 1C). However, an obvious enrichment of this bacterium was revealed in advanced stage tissues (III/IV, n = 20; *P* = 0.003, Figure 1D).

### The increased burden of intratumoral *F. nucleatum* predicts poor prognosis in locally advanced stage cervical cancer

The median follow-up time for patients in our study was 60 months (range from 0.5 to 148 months). During the duration of the follow-up period, there were 18 patients (16.1%) who died, and there were 22(19.6 %) patients who were noted to have had progression and further development of tumors. Over this same period, the median OS was 60.5 months and the median PFS was 57.4 months. Based on observed levels of *F. nucleatum*, patients were divided into two groups derived from a cut-off value (ΔΔ*Ct*=1.06) inferred from the receiver operating characteristic curve (ROC curve) which corresponded to a measure of burden of *F. nucleatum*. The cutoff gives important information for the levels of the highest sensitivities and specificities that were accurate when used to predict cervical cancer survival rates.

**Figure 2:**
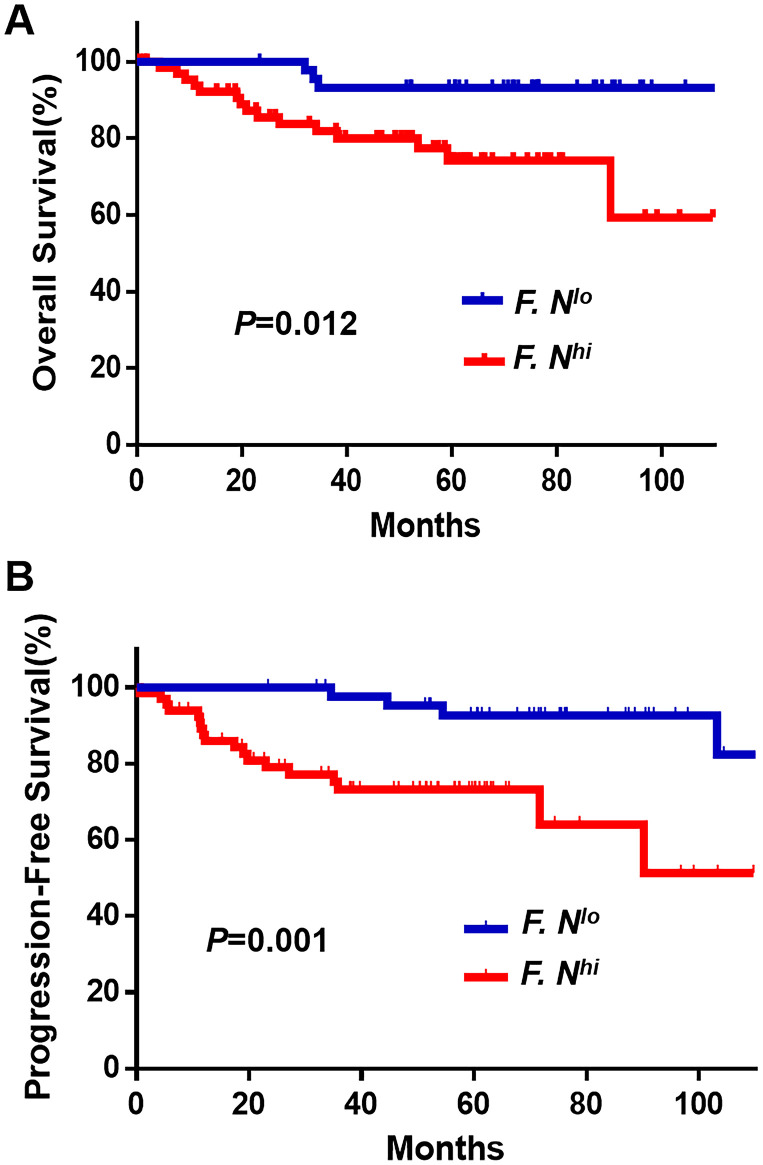
**Cumulative survival curves of *F. nucleatum* for cervical cancer patients.** Patients, divided into two groups derived from a cut-off value (ΔΔ*Ct* = 1.06), with higher *F. nucleatum* (n = 67) burdens have shorter OS (**A**) and PFS (**B**) than whom in lower group (n = 45). The OS and PFS curves were generated by the Kaplan–Meier method and analyzed using the log-rank test.

We next assessed *F. nucleatum* infiltration abundances for the 112 patients with cervical cancer and summarized their clinicopathological characteristics in Table 1. Kaplan-Meier analyses indicated that the relationships between *F. nucleatum* burdens and survival rates was dependent upon *F. nucleatum* infiltration such that patients with high levels exhibited poorer OS (*P* = 0.012, Figure 2A) compared with results for the low-burden group. Similarly, this finding was subsequently confirmed in progression-free survival analyses (*P* = 0.001, Figure 2B). These determinations allowed us to answer the question of whether or not the presence of *F. nucleatum* influenced patient survival rates.

**Table 1:** Clinicopathological characteristics of patients with cervical cancer.

**Characteristics**		**No. of patients**	***%***
*Age, years*			
>35		85	75.9
≤35		27	24.1
*FIGO stage*			
I(IB1, IB2)		84	75.0
II(IIA, IIB)		28	25.0
*Grade of differentiation*			
1		6	5.4
2		37	33.0
3		69	61.6
*Greatest tumor dimension, cm*			
>4cm		87	77.7
≤4cm		23	20.5
*Lymphovascular space invasion*			
Yes		6	5.4
No		106	94.6
*Depth of cervical invasion*			
≥ 66%		48	60.8
<66%		31	39.2
*Uterine corpus invasion*			
Yes		59	52.7
No		52	46.4
*Pelvic lymph node metastasis*			
Yes		22	20.0
No		88	80.0
Vital status(at follow-up)			
Death		18	16.1
Alive		94	83.9
*Distant metastasis and recurrence*			
Yes		22	19.6
No		90	80.4

### High intratumoral burdens of *F. nucleatum* can be viewed as a survival independent risk factor for recurrence

We investigated the clinical significance of *F. nucleatum* levels in the context of other clinicopathological features via univariate and multivariate analysis. In Table 2, univariate Cox regression OS analysis indicated patients with depth of cervical invasion (HR = 11.4; 95%CI = 1.5-86, P = 0.019), poor tumor differentiation (HR = 6.5; 95%CI = 1.5-28.4, P = 0.013) or high *F. nucleatum* level (HR = 4.9; 95%CI = 1.4-17.4, P = 0.013) were associated with shorter OS. However, *F. nucleatum* level had no significant differences in its multivariate Cox model. Furthermore, in term of patient PFS survival in Table 3, the univariate Cox regression analysis showed that cervical cancer patients with Pelvic lymph node metastasis (HR = 2.9; 95%CI = 1.2-6.8, P = 0.014), poor tumor differentiation (HR = 4.5; 95%CI = 1.5-13.6, P = 0.008) or high *F. nucleatum* level (HR = 5.2; 95%CI = 1.7-16.1, P = 0.003) were associated with shorter PFS. Next, a multivariate Cox model was built to analyses these factors which were in keeping with above univariate analysis and revealed that Pelvic lymph node metastasis (HR = 3.1; 95%CI = 1.1-8.8, P = 0.029), and those with poor tumor differentiation (HR = 4.8; 95%CI = 1.2-18.3, P = 0.023), and those higher *F. nucleatum* level (HR = 4.8; 95%CI = 1.2-18.6, P = 0.024) were associated with an increased risk of tumor progression. We concluded this bacterium was indeed an independent risk factor for predicting poor PFS.

**Table 2:** Univariate and multivariate analysis of factors associated with overall survival in cervical cancer patients^a^.

**Variables**	**Subset**	**HR ^b^ (95%CI)**	***P*-value**
**Univariate analysis**			
Age, years	>35 *vs.* ≤35	0.6(0.2-1.6)	0.330
FIGO stage	IIA *vs*.IB1/IB2	1.6(0.6-4.4)	0.318
Greatest tumor dimension, cm	>4 *vs.* ≤4	2.1(0.8-5.7)	0.141
Lymphovascular space invasion	Yes or no	1.8(0.4-8.2)	0.404
Depth of cervical invasion	≥66% *vs.* <66%	11.4(1.5-86)	**0.019** ^a^
*Uterine corpus invasion*	Yes or no	1.7(0.9-3.2)	0.102
*Pelvic lymph node metastasis*	Yes or no	1.5(0.5-4.2)	0.428
Differentiation	poor *vs.* well/moderate	6.5(1.5-28.4)	**0.013** ^a^
*F. nucleatum level*	high *vs.* low	4.9(1.4-17.4)	**0.013** ^a^
**Multivariate analysis**			
Differentiation	poor *vs.* well/moderate	6.1(1.2-29.7)	**0.023** ^a^
Lymphovascular space invasion	Yes or no	7.1(1.0-50.1)	**0.047** ^a^
Depth of cervical invasion	≥66% *vs.* <66%	16.7(1.9-148)	**0.011** ^a^

^a^Cox proportional hazards regression model. Variables used in multivariate analysis were adopted by univariate analysis. Significant *p* values (< 0.05) are shown in bold font.

^b^HR > 1, risk for death increased; HR < 1, risk for death decreased.

Abbreviations: HR, hazard ratio; CI, confidence interval; NA, not applicable.

**Table 3:** Univariate and multivariate analysis of factors associated with progression-free survival in cervical cancer patients^a^.

**Variables**	**Subset**	**HR(95%CI)**	***P*-value**
**Univariate analysis**			
Age, years	>35 *vs.* ≤35	0.4(0.2-1.0)	0.055
FIGO stage	IIA/IIB *vs*.IB1/IB2	1.2(0.5-3.2)	0.649
Greatest tumor dimension, cm	>4 *vs.* ≤4	0.6(0.2-2.1)	0.476
Lymphovascular space invasion	Yes or no	1.5(0.3-6.3)	0.591
Depth of cervical invasion	≥66% *vs.* <66%	2.8(0.9-8.7)	0.067
Uterine corpus invasion	Yes or no	1.9(0.8-4.6)	0.137
Pelvic lymph node metastasis	Yes or no	2.9(1.2-6.8)	**0.014** ^a^
Differentiation	poor *vs.* well/moderate	4.5(1.5-13.6)	**0.008** ^a^
*F. nucleatum level*	high *vs.* low	5.2(1.7-16.1)	**0.003** ^a^
**Multivariate analysis**			
Pelvic lymph node metastasis	Yes or no	3.1(1.1-8.8)	**0.029** ^a^
Differentiation	poor *vs.* well/moderate	4.8(1.2-18.3)	**0.023** ^a^
*F. nucleatum level*	high *vs.* low	4.8(1.2-18.6)	**0.024** ^a^

### The high levels of local *F. nucleatum* were correlated with tumor differentiation

**Figure 3:**
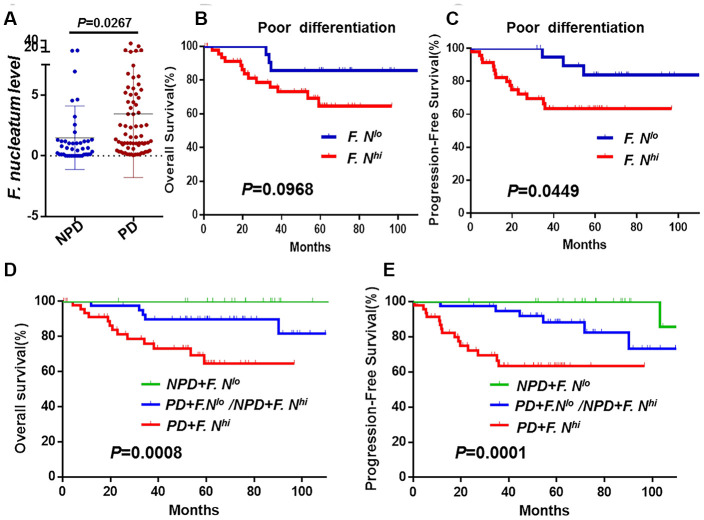
**The association between *F. nucleatum* levels and tumor histological differentiation.** (**A**) *F. nucleatum* burdens in 112 tumors tissues. The mean amount of *F. nucleatum* was increased in poor (n = 69) differentiation tumors compared with well (n = 6) or moderate (n = 37) differentiation tumors. Data are expressed as mean±SD (bars); Kaplan-Meier analysis of OS (**B**) and PFS (**C**) for patients with high (red, n = 48) or low (blue, n = 21) *F. nucleatum* levels in poor differentiation (PD) cancer tissues; Kaplan-Meier analysis of OS (**D**) and PFS (**E**) for patients with poor differentiation and high *F. nucleatum* levels (red, n = 47, PD+*F. nucleatum^hi^*) vs. poor differentiation and low *F. nucleatum* levels or non-poor differentiation(NPD, well/moderate differentiation) and high *F. nucleatum* levels (blue, n = 40, PD+*F. ^lo^*/NPD*+F. ^hi^*) vs. NPD and low *F. nucleatum* levels (green, n = 24, NPD+*F. nucleatum^lo^*). Differences were assessed with an unpaired two-tailed t-test. The OS and PFS curves were generated by the Kaplan–Meier method and analyzed using the log-rank test.

The relationship between the patients’ intratumoral *F. nucleatum* status and clinicopathological characteristics was investigated in 112 patients. Table 4 suggested that levels of *F. nucleatum* and histological differentiation (*P* = 0.007), recurrence (*P* = 0.015) and vital status (*P* = 0.022) were obviously correlated. Patients with poor differentiation tended toward a higher burden of *F. nucleatum.* Notably, we measured of *F. nucleatum* abundances based upon their differentiation. This assessment demonstrated that there was an evident enrichment of *F. nucleatum* in tissues afflicted with cervical cancer with poor differentiation compared to samples of low pathological grade tissues. It can be seen in Fig 3A that *F. nucleatum* burdens were confirmed to have been associated with differentiation such that women with higher pathological grading tended toward having higher measures of abundances (poor vs. non-poor differentiation also known as well/moderate differentiation; *P* = 0.0267, Figure 3A). The assessment also revealed that there were no significant effects of patient age, FIGO stage, tumor dimension, lymphovascular space invasion, depth invasion, uterine corpus invasion or pelvic lymph node metastasis on the associated levels of *F. nucleatum* in locally advanced cancer tissues.

**Table 4:** Associations between *F. nucleatum level* and clinicopathological characteristics in cervical cancer.

**Variables**	***F. nucleatum level***		
	**Low**	**High**	***P***
Age, years			0.145
>35	37(43.5)	48(56.5)	
≤35	8(29.6)	19(70.4)	
FIGO stage			0.241
IIA/IIB	13(48.1)	14(51.9)	
IB1/IB2	32(38.1)	52(61.9)	
Greatest tumor dimension, cm			0.521
>4	9(39.1)	14(60.9)	
≤4	36(41.4)	51(58.6)	
Lymphovascular space invasion			0.459
Yes	3(50.0)	3(50.0)	
No	42(39.6)	64(60.4)	
Depth of cervical invasion			0.135
≥66%	16(33.3)	32(66.7)	
<66%	15(48.4)	16(51.6)	
Uterine corpus invasion			0.065
Yes	19(32.2)	40(67.8)	
No	25(48.1)	27(51.9)	
Pelvic lymph node metastasis			0.327
Yes	10(45.5)	12(54.5)	
No	33(37.5)	55(62.5)	
Differentiation ^b^			**0.007** ^a^
Poor	21 (30.4)	48(69.6)	
Well+moderate	24(55.8)	19(44.2)	
Recurrence			**0.015** ^a^
Yes	4(18.2)	18(81.8)	
No	41(45.6)	49(54.4)	
Vital status(at follow-up)			**0.022** ^a^
Death	3 (16.7)	15(83.3)	
Alive	42(44.7)	52(55.3)	

^a^*χ^2^* test. Significant *P-*values (<0.05) are shown in bold font.

We were curious to analyses whether this bacterium had any effect on patient survival stratified by pathological grade. We still found that patients with high burden of *F. nucleatum* hada shorter PFS (*P* = 0.04, Figure 3C) in poor differentiation group, but there was no difference in OS (*P* = 0.09, Figure 3B). Importantly, when the pathological grade was combined together with the *F. nucleatum* levels, patients with high levels of this bacterium were observed worse OS (*P* = 0.0008, Figure 3D) and PFS (*P* = 0.0001, Figure 3E).

### With high intratumoral *F. nucleatum* burdens, cervical cancer cells possess the characteristics of cancer stem cells (CSCs)

**Figure 4:**
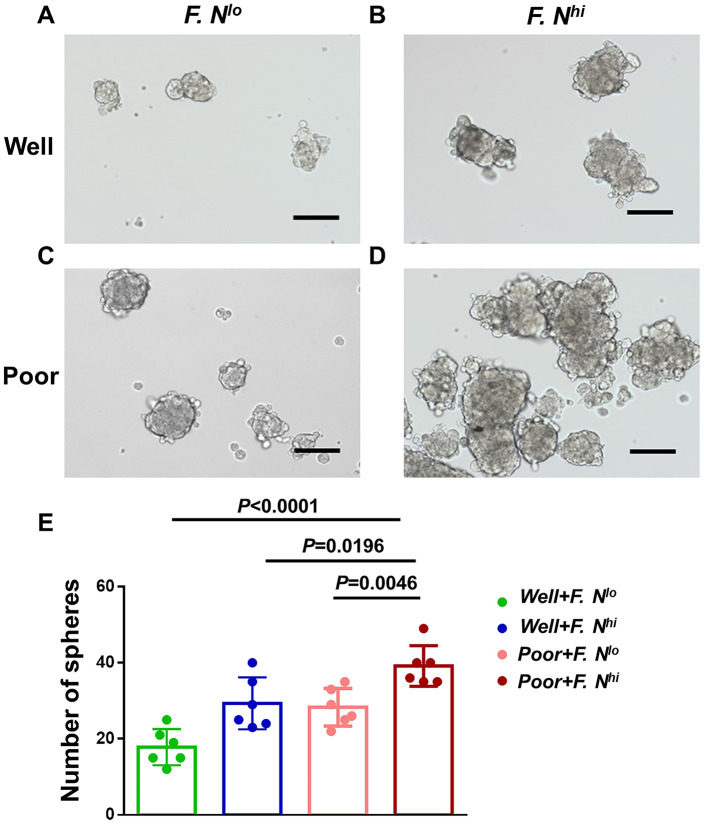
**High intratumoral *F. nucleatum* burden is associated with cervical cancer cells stemness.** Spheres in primary cervical cancer cells sorted for differentiation and intratumoral *F. nucleatum* levels- Representative Sphere formation capability for cells from patients with well differentiation and low (**A**) or high (**B**) amounts of *F. nucleatum*, or patients with poor differentiation and low (**C**) or high (**D**) levels of intra-tumor *F. nucleatum.* (**E**) Statistical analyses of Spheres for above patients. Scale bar = 200μm. Data are expressed as mean±SD (bars); Primary cells were obtained from cervical cancer patients and spheres were formed by culturing 3*10^3^ cells in 3 wells. The number of spheres (>75μm) was counted respectively. All experiments were performed in triplicate.

Given our data suggested that the level of intra-tumor *F. nucleatum* are correlated with the differentiation of cervical cancer cells, we further aimed to explore whether cancer cells obtained from patients with high intratumoral *F. nucleatum* levels were more stem-like. Primary cervical cancer cells were cultivated from patients with high or low intratumoral *F. nucleatum* levels, and poor or well differentiation (n = 6 respectively; Figure 4A–4D). Sphere formation assay was used to survey their self-renewal capacity. It indicated that more significantly larger spheres were formed by cells from high intratumoral *F. nucleatum* levels, especially in patients with poor differentiation at the same time (*P*<0.0001, Figure 4E).

**Figure 5:**
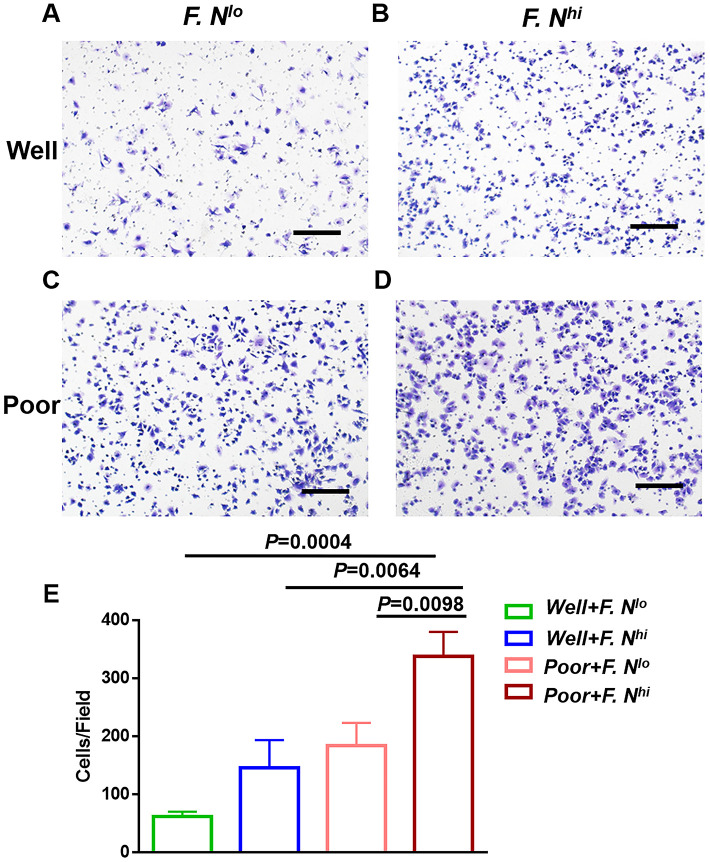
**High levels of intratumoral *F. nucleatum* are associated with cancer cells invasion capacity.** Representative Matrigel transwell for cells sorted for well differentiation and low (**A**) or high (**B**) amounts of *F. nucleatum*, or poor differentiation and low (**C**) or high (**D**) levels of intra-tumor *F. nucleatum.* (**E**) Statistical analyses of invasion cells for these groups. Scale bar = 500μm. Data are expressed as mean±SD (bars). All experiments were performed in triplicate.

CSCs have a tendency to metastasize and our data suggested that *F. nucleatum* was a prognostic indicator of recurrence and metastasis in patients with cervical cancer, then the metastatic capacity of tumor cells was further examined using Matrigel invasion assays, wherein *F. nucleatum* levels were shown to increase the invasive capacity of primary cervical cancer cells (Figure 5D vs. 5C), even in patients with well differentiation (Figure 5B vs. 5A). In total, both sphere-formation and invasive abilities were significantly increased when *F. nucleatum* levels stood high (Figures 4E, 5E).

**Figure 6:**
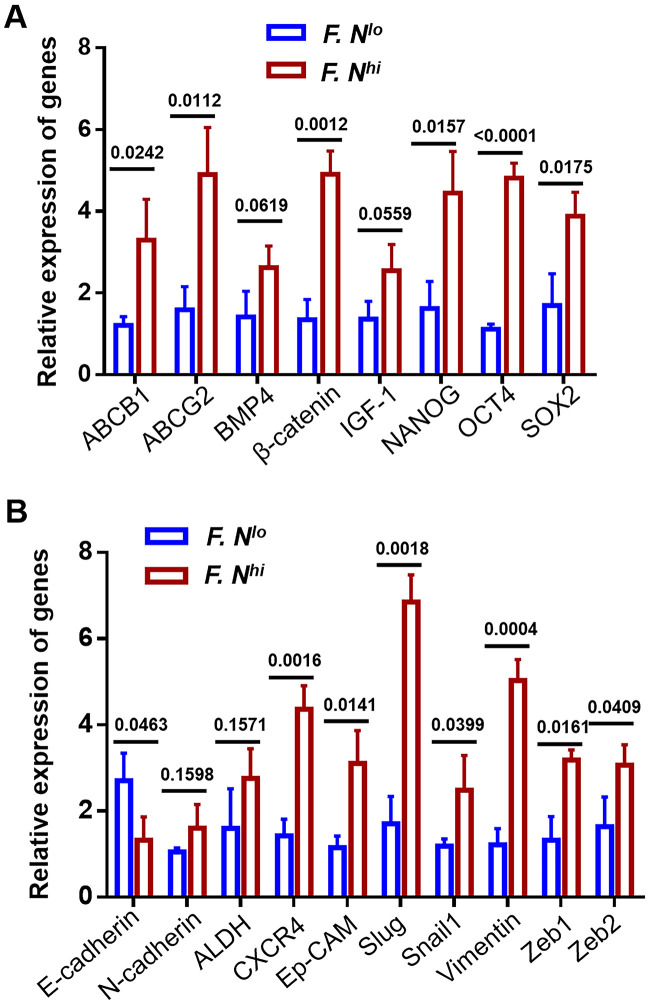
**The expression levels of known CSCs and metastasis-related genes.** Expression of 8 CSCs regulated genes (**A**) and 10 metastasis associated proteins (**B**) were compared by quantitative RT-PCR between cells obtained from patients with low and high intra-tumor *F. nucleatum* infiltration. Error bars represent the mean±SD of three independent experiments.

Finally, to confirm that high level of intratumrol *F. nucleatum* was functionally active, the expression levels of known genes related to tumor stemness (Figure 6A) and invasion (Figure 6B) were detected by RT-qPCR between patients with high and low *F. nucleatum* levels. We observed that expression levels of most genes were significantly increased in primary cervical cancer cells obtained from patients with high intratumoral *F. nucleatum* levels.

## DISCUSSION

In this study, we made several novel and seemingly important observations. For example, we provided the first line of evidence for measures of the clinical significance of *F. nucleatum as* a potential prognostic and predictive biomarker for cervical cancer. *F. nucleatum* is a likely independent risk factor that has high value for use in predicting poor PFS. Further, we observed that there were significant differences in the status of *F. nucleatum* between assessments of primary cervical cancer afflicted tissues and corresponding recurrence. Higher burdens of this bacterium were present in patients’ cervical cancer recurrent tissue compared with their primary cancer tissues. Additionally, we illustrated that cervical cancer cells from patients with relatively high intratumoral levels of *F. nucleatum* were more ‘stem-like’, which is an important finding that might help to improve treatments and treatment outcomes for afflicted patients.

It has been reported that abnormal types, compositions, and abundances of vaginal microbiota plays an important role in the development of cervical carcinoma [21]. There are stages preceding cervical cancer onset during which cervical and vaginal microenvironments are modified, such as including changes in measures of vaginal acidity and in the patterns of cytokines. Such modifications eventually can lead to a localized immunosuppressive state [22]. In the vaginal microenvironment, factors such as the presence of *Lactobacilli* spp., a low vaginal pH (<4.5), and antimicrobial peptides are part of the defense mechanisms present. When there is an imbalance of these elements and of the vaginal microenvironment defense system, physicochemical changes arise and produce histological alterations of cervical epithelium and vaginal mucosa. These are conditions that exert selective pressures on associated microbiota caused the dysbiosis of vaginal microbiota [23–25]. Most of research which examined the female genital tract microbiome has been carried out at the vaginal level and with respect to tumorigenesis, thus far, few studies have involved the cervical epithelium and intratumor cervical cancer microbiota. In our study, we provided novel information on the potential for intraumoral bacteria help predict prognoses for female patients afflicted with tumors in the genital tract. Ultimately, these results suggest that intraltumral *F. nucleatum* promotes tumor aggression and impacts patients’ prognosis in cervical cancer.

Moreover, current studies focused on primary tumor tissues and did not identify or examine aspects related to the presence of *F. nucleatum* levels in the paired tumor recurrent tissues. We collected paraffin sections of paired primary tumor tissues, recurrent tumor tissues and adjacent tumor tissues from locally advanced cervical cancer patients those whom were found to have a recurrence of cervical cancer. Although most of the detection rates in frozen tissues are generally higher vs. FFPE tissues, we finally confirmed that this bacterium had a significantly higher abundance in recurrent cervical cancer tissues than primary tumor. Results from Yamamura indicated that *F. nucleatum* levels were significantly higher in esophageal squamous carcinoma patients with advanced stages of the disease compared to patients that were still in the early stages of the disease (T1 vs. T2-4) [20]. In our research, there was no effect of FIGO stage I-II on the infiltration of *F. nucleatum* in the cancer tissues, but its burdens would be increased sharply in advanced cancer (I-II vs. III-IV). It's not hard to speculate reasonably, *F. nucleatum* enrichments increase the risk of cervical cancer progression or recurrence.

Despite our study found that *F. nucleatum* levels influence cancer progression or recurrence and are associated with the stemness of cervical cancer cells, there are certain limitations. The dysbiosis of intratumor microbiota has been implicated in tumor development and can play a large role in influencing treatment outcomes for patients with cancers [26, 27]. But the mechanism by which this bacterium works needs to be elaborated. Although not explicitly stated, some studies have implied that this bacterium is involved in the regulation of tumor stem cells. For example, *F. nucleatum* activates autophagy-related pathways in colorectal cancer through modulation of TLR4 and MYD88 innate signaling, along with certain miRNAs which subsequently promote chemoresistance [28]. *F. nucleatum* infection increases BIRC3 via the TLR4/NF-κB pathway in CRC cells, and further reduced the chemosensitivity of CRC cells to 5-Fu [29]. Reports from Rubinstein indicated that *F. nucleatum* expresses FadA adhesin protein on the bacterial surface, which increasesAnnexin A1 expression, a modulator of Wnt/β-catenin signaling, through E-cadherin. Also, FadA in known to further bind to E-cadherin, thereby activating β-catenin signaling, which can ultimately promotes tumor development [30, 31]. We further detect the levels of known CSCs and metastasis-related genes [32, 33], and our data imply that high level of intra-tumor *F. nucleatum* may induce stemness through activating certain specific transcription factors, such as NANOG, OCT4, SOX2 and activate associated signaling pathway, such as WNT/β-catenin and IGF-1 receptor pathway. Therefore, increased *F. nucleatum* burden may next activate many other sequences of metastasis, such as CXCR4, Ep-CAM, Slug, Snail1 and Zeb1/2. In combination with our findings, these results suggest that there is a plausible role of the *F. nucleatum* in the dynamics underlying cervical cancer, while whether other novel and effective mechanisms existing merits further investigation.

## CONCLUSIONS

We identified that previously unreported cervical cancer-associated and localized *F. nucleatum* levels are higher. High intratumor levels of *F. nucleatum* were found to have been correlated with poor OS and PFS rates for afflicted patients and can be used as effective independent prognostic factors for forecasting patient PFS rates. Furthermore, our study demonstrated that there were *F. nucleatum*-related differences in intratumor profiles of cervical cancer for comparisons between primary cancer tissues and recurrent cancer tissues. Importantly, cervical cancer cells, obtained from patients with high intratumoral *F. nucleatum* burdens, probably possess the characteristics of CSCs. Additional research is still needed to validate our findings and should be undertaken using larger cohorts and such as to determine the biological significance and mechanisms of these observed differences.

## MATERIALS AND METHODS

### Participants and clinical data

This study was randomly enrolled a total of 112 patients diagnosed with stage IB1/IB2-IIA1/IIA2 squamous carcinoma of the cervix who underwent surgical resection at the Sun Yat-Sen University Cancer Center and postoperative chemotherapy or radiation if pathologic risk factors are discovered between 2010 and 2015. Additionally, we followed and collected paraffin sections of paired primary tumor, recurrent tumor tissues (stump recurrence or recurrence confined to the pelvis) and adjacent tumor tissues from 23 patients those with IB1/B2-IIA stage, and other 20 patients those with stage III- IV who underwent cervical biopsy between 2016 and 2018 at the Guangdong Provincial People’s Hospital and Sun Yat-Sen University Cancer Center. None of the patients underwent anti-cancer therapies before surgery, and no histologically confirmed serious complications or other malignant diseases had been reported. The clinicopathological characteristics of the patients are summarized in Table 1. Tumor stages were determined according to the classification system of the International Federation of Gynecology and Obstetrics (FIGO 2009) classification guidelines. All patients attended follow-up visits at the outpatient clinic with regular surveillance for recurrence via recording symptom and physical examination, the serum squamous cell carcinoma antigen level, chest radiography, pelvic and abdominal ultrasonography at 3- to 6- month intervals. When recurrence or metastasis was suspected, further examinations, like CT and PET-CT scan were performed. Biopsies were taken when it is necessary. Overall survival (OS) was defined as the interval between surgery and death or the last follow-up. Progression-free survival (PFS) was the date of surgery to recurrence, the last observation for patients without recurrence, or death if no recurrence was observed.

### RNA extraction and real-time qRT-PCR

Total RNA from primary cervical cancer cells was extracted by Trizol reagent (Life Technology). Reverse transcriptase reactions by MMLV reverse transcriptase reagents (Promega, Madison, USA) were performed following manufacturer’s protocol. Gene expression levels were normalized to house-keeping gene β-actin. Reactions were performed in triplicate with the Roche LightCycler 480 II PCR system (Roche Diagnostics, Rotkreuz, Switzerland). Primer sequences are listed in Supplementary Table 1.

### DNA extraction and qPCR assays

Genomic DNA from 112 locally advanced patients’ fresh frozen tissues were extracted using DNeasy Power Soil Kit (12888-100, Qiagen, Dusseldorf, Germany). Genomic DNA from the formalin-fixed paraffin-embedded (FFPE) tissues was extracted using the QIAamp DNA FFPE Tissue Kit (56404-50, Qiagen, Frankfurt, Germany). The amount of *F. nucleatum* DNA was quantified by use of a qPCR assay, the *nus* G gene and the reference human gene SLCO2A1 were amplified using custom TaqMan primer sets (Promega, Madison, WI, USA) in 384-well PCR plates, as described previously [17].

### Primary cells obtained and sphere formation

We used the cancer tissue-originated spheroid method for the primary culture of cervical cancer cells by Hiroko Endo et al with minor modifications [34]. We minced surgically resected primary cervical tissues with a scalpel into approximately 1-mm^3^ pieces, and washed in Hank’s balanced salt solution (HBSS). Specimens were transferred to a 50-ml centrifuge tube and digested 30 minutes in 37° C by 50μg/ml collagenase and 1% PenStrep. Digestion products were passed through 500 and 250-μm metal mesh filters to remove large masses of undigested and then filtered through 100 and 40-μm cell strainers (BD, Franklin Lakes, USA). Cells in the flow-through fractions were adjusted to a 10^6^ cells/ml concentration in sorting buffer (1× PBS), and and cultured in medium of serum-free DMEM/F12 had to contain certain key component supplementing with B27 (1:50; Invitrogen, California, USA), 20ng/ml epidermal growth factor (EGF; 1:5000; R&D Systems), 20ng/ml basic fibroblast growth factor (bFGF; 1:5000; R&D Systems) for a total of 7 days.

### Cancer cells invasion assays

For invasion assays, 10^5^ cells were plated in top transwell chambers (BD, Massachusetts, USA) coated on the inside with 1:4 diluted Matrigel (BD, Bedford, USA) in the insert of a 24-well culture plate. Medium containing 10% fetal bovine serum was added to the wells outside of chamber as a chemoattractant. After 48h incubator, cells inside the chamber were gently removed with a cotton swab. Invasion cells located on the lower side of the chamber were stained with crystal violet, air dried and photographed. Three independent experiments were performed and data are presented as the mean ± standard deviation.

### Statistical analysis

Statistical analysis was performed by IBM SPSS Soft 21 (IBM Corporation; United States) and GraphPad Prism 6 (GraphPad Software; United States). Continuous variables were showed as medians and compared by a t-test or Mann-Whitney U test. We used Pearson’s χ ^2^ test or Fisher’s exact test to examine the relationship between *F. nucleatum* levels expression and clinicopathological characteristics as appropriate. Survival estimates were calculated using the Kaplan-Meier analysis and compared via log-rank test. Prognostic parameters with effects on survival in univariate analysis were included in a multivariate Cox proportional hazards regression model. All *P*-values were evaluated using two-sided tests, and *P*-values of <0.05 were considered statistically significant.

**Ethics approval**

This study conformed strictly to the ethical guidelines of the Declaration of Helsinki and was approved by the Research Ethics Committee of Guangdong Provincial People’s Hospital and Sun Yat-Sen University Cancer Center. A written informed consent was obtained from all patients.

## Supplementary Material

Supplementary Table 1
