# Supplementary material for: Intratumoral levels and prognostic significance of Fusobacterium nucleatum in cervical carcinoma
Source: Aging (Albany NY). 2020 Nov 14;12(22):23337–50. doi: 10.18632/aging.104188 (PMC7746363; doi:10.18632/aging.104188)
Supplement: Supplementary Table 1 [file aging-12-104188-s001..pdf]

SUPPLEMENTARY TABLE

Supplementary Table 1. Primers for real-time PCR.

Genes	Forward	Reverse
β-actin	5'-TCCCTGGAGAAGAGCTACGA-3'	5'-AGCACTGTGTTGGCGTACAG-3'
ABCB1	5'-GGGATGGTCAGTGTTGATGGA-3'	5'-GCTATCGTGGTGGCAAACAATA-3'
ABCG2	5'-TGAGCCTACAACTGGCTTAGA-3'	5'-CCCTGCTTAGACATCCTTTTCAG-3'
BMP4	5'-GGGAAACTTTCGATGTGAGC-3'	5'-AACGATCGGCTGATTCTGAC-3'
FGF	5'-AGAAGAGCGACCCTCACATCA-3'	5'-CGGTTAGCACACACTCCTTTG-3'
IGF1	5'-GCTCTTCAGTTCGTGTGTGGA-3'	5'-GCCTCCTTAGATCACAGCTCC-3'
NANOG	5'-TCTTCCTGGTCCCCACAGTTT-3'	5'-GCAAGAATAGTTCTCGGGATGAA-3'
OCT4	5'-CGAAAGAGAAAGCGAACCAG-3'	5'-AACCACACTCGGACCACATC-3'
SOX2	5'-ACACCAATCCCATCCACACT-3'	5'-CCTCCCCAGGTTTTCTCTGT-3'
E-cadherin	5'-AACAACTGCATGAAGGCGGGAATC-3'	5'-CCTGTGCAGCTGGCTCAAATCAAA-3'
N-cadherin	5'-GGCTGAAAATAGACCCCGTG-3'	5'-GCTGTGATGTTAATTGAGTTGGG-3'
ALDH	5'-TTGGAATTTCCCGTTGGTTA-3'	5'-CTGTAGGCCCATAACCAGGA-3'
β-catenin	5'-ACAACTGTTTTGAAAATCCA-3'	5'-CGAGTCATTGCATACTGTCC-3'
Ep-CAM	5'-GCTGGCAACAAGTTGCTCTCTGAA-3'	5'-CGTTGCACTGCTTGGCTTTGAAGA-3'
SLUG	5'-CTGGGCGCCCTGAACATGCAT-3'	5'-GGCTTCTCCCCCGTGTGAGTTCTA-3'
SNAIL1	5'-TGCGCTACTGCTCGGCGAAT-3'	5'-AGGGCTGCTGGAAGGTAAACTCTGG-3'
Vimentin	5'-GAGAACTTTGCCGTTGAAGC-3'	5'-GCTTCCTGTAGGTGGCAATC-3'
Zeb1	5'-AGTGGTCATGATGAAAATGGAACACCA-3'	5'-AGGTGTAACTGCACAGGGAGCA-3'
Zeb2	5'-GACAGATCAGCACCAAATGC-3'	5'-GCTGATGTGCGAACTGTAGG-3'
